# CYP2D6 phenotype, tamoxifen, and risk of contralateral breast cancer in the WECARE Study

**DOI:** 10.1186/s13058-018-1083-y

**Published:** 2018-12-10

**Authors:** Jennifer D. Brooks, Elizabeth A. Comen, Anne S. Reiner, Irene Orlow, Siok F. Leong, Xiaolin Liang, Lene Mellemkjær, Julia A. Knight, Charles F. Lynch, Esther M. John, Leslie Bernstein, Meghan Woods, David R. Doody, The WECARE Study collaborative group, Jonine L. Bernstein, Marinela Capanu, Xiaolin Liang, Irene Orlow, Anne S. Reiner, Mark Robson, Meghan Woods, Leslie Bernstein, John D. Boice, Jennifer D. Brooks, Patrick Concannon, Dave V. Conti, David Duggan, Joanne W. Elena, Robert W. Haile, Esther M. John, Julia A. Knight, Charles F. Lynch, Kathleen E. Malone, Lene Mellemkjær, Jørgen H. Olsen, Daniela Seminara, Roy E. Shore, Marilyn Stovall, Daniel O. Stram, Marc Tischkowitz, Duncan C. Thomas, Kristina Blackmore, Anh T. Diep, Judy Goldstein, Irene Harris, Rikke Langballe, Cecilia O’Brien, Susan Smith, Rita Weathers, Michele West, Kathleen E. Malone, Jonine L. Bernstein

**Affiliations:** 1 University of Toronto, Dalla Lana School of Public Health Sciences, 155 College St. HSB 676, Toronto, ON M5T 3M7 Canada; 2 Memorial Sloan Kettering Cancer Center, New York, NY USA; 3 Danish Cancer Society Research Center, Copenhagen, Denmark; 4 Lunenfeld-Tanenbaum Research Institute, Sinai Health System, Toronto, Canada; 5 University of Iowa, Iowa City, IA USA; 6 Department of Medicine and Stanford Cancer Institute, Stanford University School of Medicine, Stanford, CA USA; 7 Beckman Research Institute, City of Hope National Medical Centre, Duarte, CA USA; 8 Fred Hutchinson Cancer Research Center, Seattle, WA USA

**Keywords:** Contralateral breast cancer, Tamoxifen, CYP2D6

## Abstract

**Background:**

Tamoxifen treatment greatly reduces a woman’s risk of developing a second primary breast cancer. There is, however, substantial variability in treatment response, some of which may be attributed to germline genetic variation. CYP2D6 is a key enzyme in the metabolism of tamoxifen to its active metabolites, and variants in this gene have been associated with reduced tamoxifen metabolism. The impact of variation on risk of contralateral breast cancer (CBC) is unknown.

**Methods:**

Germline DNA from 1514 CBC cases and 2203 unilateral breast cancer controls was genotyped for seven single nucleotide polymorphisms, one three-nucleotide insertion-deletion, and a full gene deletion. Each variant has an expected impact on enzyme activity, which in combination allows for the classification of women as extensive, intermediate, and poor metabolizers (EM, IM, and PM respectively). Each woman was assigned one of six possible diplotypes and a corresponding CYP2D6 activity score (AS): EM/EM (AS = 2), EM/IM (AS = 1.5), EM/PM (AS = 1), IM/IM (AS = 0.75), IM/PM (AS = 0.5), and PM/PM (AS = 0). We also collapsed categories of the AS to generate an overall phenotype (EM, AS ≥ 1; IM, AS = 0.5–0.75; PM, AS = 0). Rate ratios (RRs) and 95% confidence intervals (CIs) for the association between tamoxifen treatment and risk of CBC in our study population were estimated using conditional logistic regression, stratified by AS.

**Results:**

Among women with AS ≥ 1 (i.e., EM), tamoxifen treatment was associated with a 20–55% reduced RR of CBC (AS = 2, RR = – 0.81, 95% CI 0.62–1.06; AS = 1.5, RR = 0.45, 95% CI 0.30–0.68; and AS = 1, RR = 0.55, 95% CI 0.40–0.74). Among women with no EM alleles and at least one PM allele (i.e., IM and PM), tamoxifen did not appear to impact the RR of CBC in this population (AS = 0.5, RR = 1.08, 95% CI 0.59–1.96; and AS = 0, RR = 1.17, 95% CI 0.58–2.35) (*p* for homogeneity = – 0.02).

**Conclusion:**

This study suggests that the CYP2D6 phenotype may contribute to some of the observed variability in the impact of tamoxifen treatment for a first breast cancer on risk of developing CBC.

**Electronic supplementary material:**

The online version of this article (10.1186/s13058-018-1083-y) contains supplementary material, which is available to authorized users.

## Introduction

The high incidence of breast cancer in the United States, coupled with a high rate of survival, places an increasing number of women at risk of contralateral breast cancer (CBC) [1]. CBC is the most common malignancy among breast cancer survivors, accounting for nearly 40% of all second cancers [2]. Tamoxifen treatment for a first primary breast cancer greatly reduces a woman’s risk of developing CBC [3–6]. However, there is substantial variability in treatment response, some of which may be attributed to germline genetic variation in drug metabolism.

CYP2D6 is a key enzyme in tamoxifen metabolism and has been central to the pharmacogenetic investigation of tamoxifen treatment response. Focus has been placed on CYP2D6 because it is responsible for the metabolism of tamoxifen to its two primary active metabolites, 4-hydroxytamoxifen and endoxifen. Compared to tamoxifen, these metabolites have over 100-fold higher affinity for the estrogen receptor (ER), and 30-fold to 100-fold greater potency in suppressing estrogen-dependent tumor cell growth [7, 8]. Carriers of certain variants in *CYP2D6* have been shown to have reduced enzyme activity and lower circulating levels of active metabolites [9].

Given these effects on tamoxifen metabolism, studies have examined the impact of variation in *CYP2D6* on breast cancer recurrence and mortality, with mixed results [10–27]. These studies have not addressed the impact of variation in *CYP2D6* on risk of CBC. It is currently unknown whether germline variation in *CYP2D6* modifies the association between tamoxifen treatment and risk of CBC. Given the relationship between tamoxifen and risk of CBC, this is potentially a critical clinical issue. The objective of this study was to examine the impact of genetically inferred CYP2D6 phenotype on the association between tamoxifen treatment for a first breast cancer and the risk of CBC in a large population-based case–control study.

## Materials and methods

### Study population

The Women’s Environmental Cancer and Radiation Epidemiology (WECARE) Study is a multicenter, population- based case–control study where cases are women with asynchronous CBC and controls are women with unilateral breast cancer (UBC). Case–control recruitment for the WECARE Study was conducted in two phases: WECARE I (2001–2004) and WECARE II (2009–2012). Participants were identified through eight population-based cancer registries: Los Angeles County Cancer Surveillance Program; Cancer Surveillance System of the Fred Hutchinson Cancer Research Center (Seattle, WA, USA); State Health Registry of Iowa; The Cancer Surveillance Program of Orange County/San Diego—Imperial Organization for Cancer Control (Orange County/San Diego, CA, USA); the Greater Bay Area Cancer Registry (San Francisco Bay Area Region and Santa Clara Region, CA, USA); and the Sacramento and Sierra Center Registries (Sacramento Region, CA, USA). These cancer registries all contribute to the National Cancer Institute (NCI) Surveillance, Epidemiology and End Results (SEER) program in the United States. Patients were also recruited from the Ontario (Canada) Cancer Registry and the Danish Breast Cancer Cooperative Group Registry, supplemented by data from the Danish Cancer Registry. All study participants provided written informed consent, and the study protocol was reviewed and approved by the Institutional Review Board at each recruitment site and the ethical committee systems in Denmark and Ontario. Across the eight cancer registries a total of 2354 CBC cases and 3599 UBC controls were identified as being eligible and were approached for the study. The final number of participants who completed the interview and provided a biospecimen for DNA analysis was 1518 (64%) cases and 2208 (61%) controls. Reasons for nonparticipation have been described in detail [28]. An additional nine participants were excluded from the current analysis due to low quality or quantity of DNA (four cases, five controls), resulting in a final sample size of 1514 CBC cases and 2203 UBC controls.

Details of recruitment procedures, eligibility, and the study questionnaire have been described previously, and were nearly identical for the two study phases [29]. Briefly, all women were diagnosed prior to age 55 years between 1985 and 2010 with a first primary invasive breast cancer (stage I–III). Cases were diagnosed with a second primary CBC (in situ or invasive for WECARE I and invasive only for WECARE II) at least 1 year later. Controls also had no history of any second cancer diagnosis up to their reference date. The reference date for cases was the CBC diagnosis date, while for controls this was defined by adding the interval between the first breast cancer and the CBC for the matched case to the date of (first) breast cancer diagnosis for the control. Cases must also have been living in the same study reporting area for both diagnoses, while controls were required to be living in the cancer-reporting area of their (first) breast cancer diagnoses on their reference dates. Additionally, controls must not have undergone prophylactic mastectomy of the unaffected contralateral breast. Study eligibility was restricted to women who were alive when contacted and were able to provide informed consent, complete a telephone interview, and donate a blood or saliva sample for DNA extraction. Controls were matched to cases (2:1 for WECARE I and 1:1 for WECARE II) on year of birth in 5-year strata, year of diagnosis in 4-year strata, cancer registry region, and race/ethnicity. In WECARE I, cases and controls were further counter-matched based on cancer registry-reported radiation treatment such that two members of each case–control trio had received radiation treatment for their first breast cancer and the other member had not [29]. Counter-matching was not used in WECARE II; this was taken into account in all statistical analyses, as detailed in the following. The selection of case–control trios in this way for WECARE I is reflected in the frequency of radiation treatment by case–control status such that the frequencies cannot be directly compared. For this reason, weighted frequencies are provided (Table 1).

**Table 1:** Characteristics of CBC cases and UBC controls and those treated and not treated with tamoxifen from the WECARE Study population who were also screened for *CYP2D6* variants

Variable	CBC cases (*N* = 1514)	UBC controls (*N* = 2203)	Tamoxifen treatment (*N* = 1250)	No tamoxifen treatment (*N* = 2467)
Age at first diagnosis (years), median (range)	46 (24–54)	48 (24–54)	48 (24–54)	45 (23–54)
Age at reference date (years), median (range)	53 (27–73)	54 (28–73)	54 (28–73)	51 (27–71)
Length of at-risk period (years)^a^, median (range)	6.3 (1.0–19.8)	6.0 (1.0–19.3)	6.0 (1.0–19.3)	5.8 (1.0–19.8)
Study area, *N* (%)				
Iowa^b^	201 (13)	314 (14)	170 (14)	345 (14)
California^c^	655 (43)	963 (44)	634 (51)	984 (40)
Seattle^d^	223 (15)	317 (14)	249 (20)	291 (12)
Denmark^e^	279 (18)	452 (21)	57 (5)	674 (27)
Canada^f^	156 (10)	157 (7)	140 (11)	173 (7)
Year of first breast cancer diagnosis, *N* (%)				
1985–1988	238 (16)	466 (21)	122 (10)	582 (24)
1989–1992	414 (27)	643 (29)	328 (26)	729 (30)
1993–1996	425 (28)	630 (29)	353 (28)	702 (28)
1997–2008	437 (29)	464 (21)	447 (36)	454 (18)
Race/ethnicity^g^, *N* (%)				
Non-Hispanic white	1330 (88)	1971 (89)	1070 (86)	2231 (90)
Hispanic white	68 (4)	93 (4)	59 (5)	102 (4)
Black	54 (4)	75 (3)	47 (4)	82 (3)
Asian	45 (3)	52 (2)	61 (5)	36 (1)
Other	17 (1)	12 (1)	13 (1)	16 (1)
Age at menarche (years), *N* (%)				
Never had menses	3 (0)	6 (0)	1 (0)	8 (0)
< 13	722 (48)	962 (44)	622 (50)	1062 (43)
≥ 13	786 (52)	1233 (56)	625 (50)	1394 (57)
Unknown	3 (0)	2 (0)	2 (0)	3 (0)
Number of full-term pregnancies, *N* (%)				
None	320 (21)	408 (19)	246 (20)	482 (20)
1	271 (18)	340 (15)	200 (16)	411 (17)
2	556 (37)	839 (38)	444 (36)	951 (39)
3	255 (17)	386 (18)	237 (19)	404 (16)
≥ 4	107 (7)	225 (10)	118 (9)	214 (9)
Unknown	5 (0)	5 (0)	5 (0)	5 (0)
Menopausal status at first diagnosis^h^, *N* (%)				
Premenopausal	1119 (74)	1669 (76)	865 (69)	1923 (78)
Postmenopausal	387 (26)	520 (24)	377 (30)	530 (21)
Unknown	8 (1)	14 (1)	8 (1)	14 (1)
First-degree family history of breast cancer, *N* (%)				
No	999 (66)	1697 (77)	901 (72)	1795 (73)
Yes	496 (33)	466 (21)	325 (26)	637 (26)
Adopted/unknown	19 (1)	40 (2)	24 (2)	35 (1)
Stage of first primary breast cancer, *N* (%)				
Local	1059 (70)	1436 (65)	747 (60)	1748 (71)
Regional	446 (29)	756 (34)	493 (39)	709 (29)
Unknown	9 (1)	11 (1)	10 (1)	10 (0)
Histology of first diagnosis, *N* (%)				
Nonlobular	1334 (88)	1978 (90)	1061 (85)	2251 (91)
Lobular	179 (12)	222 (10)	186 (15)	215 (9)
Unknown	1 (0)	3 (0)	3 (0)	1 (0)
ER status of first diagnosis, *N* (%)				
Positive	793 (52)	1248 (57)	1047 (84)	994 (40)
Negative	467 (31)	560 (25)	129 (10)	898 (36)
Other/unknown^i^	254 (17)	395 (18)	74 (6)	575 (23)
Chemotherapy for first diagnosis, *N* (%)				
No	696 (46)	919 (42)	485 (39)	1130 (46)
Yes	818 (54)	1284 (58)	765 (61)	1337 (54)
Radiation treatment for first diagnosis^j^, *N* (%)				
WECARE I				
No	362 (51)	265 (50)	139 (25)	488 (32)
Yes	346 (49)	1130 (50)	424 (75)	1052 (68)
Unknown	0 (0)	0 (0)	0 (0)	0 (0)
WECARE II				
No	275 (34)	258 (32)	195 (28)	338 (36)
Yes	531 (66)	549 (68)	491 (71)	589 (64)
Unknown	0 (0)	1 (0)	1 (0)	0 (0)
Hormone treatment for first diagnosis, *N* (%)				
None	960 (63)	1263 (57)	NA	NA
Tamoxifen	465 (31)	785 (36)		
Other hormonal treatment^k^	89 (6)	153 (7)		
Unknown	0 (0)	2 (0)		

*CBC* contralateral breast cancer, *UBC* unilateral breast cancer, *WECARE* Women’s Environmental Cancer and Radiation Epidemiology, *ER* estrogen receptor, *NA* not available

^a^Beginning at least 1 year after first diagnosis and extending to the date of CBC diagnosis of cases

^b^The State Health Registry of Iowa

^c^Four study centers: Los Angeles County Cancer Surveillance Program; The Cancer Surveillance Program of Orange County/San Diego-Imperial Organization for Cancer Control; Greater Bay Area Cancer Registry (San Francisco Bay Area Region and Santa Clara Region); and Sacramento and Sierra Center Registries (Sacramento Region)

^d^Cancer Surveillance System of the Fred Hutchinson Cancer Research Center

^e^The Danish Breast Cancer Cooperative Group Database supplemented by the Danish Cancer Registry

^f^The Ontario Cancer Registry

^g^‘Asian’ includes Japanese, Chinese, and Filipino; ‘Other’ includes other Asian as well as all other races/ethnicities

^h^Women were classified as premenopausal if they reported having menstrual periods or being pregnant within 2 years of initial diagnosis

^i^‘Other/unknown’ category consists of women where no laboratory test was given, the test was given and the results are unknown, or the test was given and the results were borderline; estimates not reported. Start date for ER reporting in Surveillance Epidemiology and End Results was January 1, 1990

^j^Proportion of individuals treated and not treated with radiation. In WECARE 1, cases and controls were counter-matched based on cancer registry reported radiation treatment such that two members of each case–control trio had received radiation treatment for their first breast cancer diagnosis. Proportions for controls in WECARE 1 are weighted to reflect this selection. Proportions for cases in WECARE 1 (because all cases were included) and both cases and controls in WECARE II (because counter-matching was not used in WECARE II) are not weighted

^k^Other hormonal therapies include: raloxifene/Evista, tormifene/Fareston, anastrozole/Arimidex, letrozole/Femara, aromasin/Exemestane, aminoglutethimide/Cytradren, gosereline/Zoladex, leuprolide/Lupron, faslodex/Fulvestrant, and megestrol acetate/Megace

### Data collection

WECARE Study participants were interviewed by telephone using a structured questionnaire that was designed to obtain information about events occurring before the diagnosis of the first primary breast cancer, as well as events that occurred during the at-risk period. The at-risk period was defined as beginning at least 1 year after diagnosis with a first breast cancer and ending at the second diagnosis in CBC cases, or the corresponding reference date for UBC controls. The study questionnaire included questions about personal demographics, medical history, family and reproductive history, hormone use, body size, smoking status, and alcohol intake. Additionally, medical records, pathology reports, and hospital charts were used to collect detailed treatment information (i.e., chemotherapy, hormonal therapy, and radiation therapy) for the first primary breast cancer, any recurrences experienced prior to the reference date, and tumor characteristics of the first primary tumor (e.g., ER status, histology).

### Genotyping

Germline DNA was isolated from blood using standard phenol–chloroform extraction methods or from saliva using the manufacturer’s recommendations (Genotek, ON, Canada). Samples were genotyped for seven single nucleotide polymorphisms (SNPs), one three-nucleotide insertion-deletion (indel), and a full gene deletion (Table 2), accounting for most of the clinically significant variants in *CYP2D6* [30]. Genotyping was conducted in the Molecular Epidemiology Laboratory at Memorial Sloan Kettering Cancer Center using PCR-based methods.

**Table 2:** Summary of genotyped *CYP2D6* variants and associated phenotype

Allele	SNP (RefSeq)^a^	Variant	CYP2D6 activity	Activity score value^b^	Phenotype
CYP2D6*1	NA	Wild type	Normal	1	EM
CYP2D6*2	rs16947, rs1135840	2850C > T, 4180G > C	Normal	1	EM
CYP2D6*3	rs35742686	2549delA	Inactive	0	EM
CYP2D6*4	rs3892097	1846G > A,	Inactive	0	PM
CYP2D6*5	NA	Full gene deletion	Inactive	0	PM
CYP2D6*6	rs5030655	1707delT	Inactive	0	PM
CYP2D6*9	rs5030656	2615_2617del AAG	Reduced	0.5	IM
CYP2D6*10	rs1065852, rs1135840	100C > T, 4180G > C	Reduced	0.5	IM
CYP2D6*41	rs28371725	2988G > A	Reduced	0.5	IM

*SNP* single nucleotide polymorphism, *RefSeq* reference sequence, *NA* not applicable, *EM* extensive metabolizer, *PM* poor metabolizer, *IM* intermediate metabolizer

^a^Where more than one SNP is listed for a given allele, a variant at only one loci needed to be present to classify an individual as having that allele

^b^Activity score (AS) calculated as the sum of the activity score value for each allele held by an individual for a range of values from 0 to 2. One exception to this is the instance of individuals carrying two IM (reduced activity) alleles, where we provided a distinction between individuals classified as EM/PM (AS = 1), IM/IM (AS = 0.75), and IM/PM (AS = 0.5)

Variants were tested using Agena iPlex chemistry and the MassArray system (Agena Bioscience, San Diego CA, USA), with assays designed using the Agena Cx online tools (currently https://agenacx.com), and a method similar to that reported by Falzoi et al. [31], with an independent preamplification of *CYP2D6* in a long-range PCR. The PCR products were verified on a 1% agarose gel. One microliter of the amplified product was used for genotyping using specific primers and cycling conditions (Additional file 1). Products were purified enzymatically, extended and desalted, and then spotted onto 384 SpectroCHIP bioarrays (Agena Bioscience). Cluster plots were evaluated with the TyperAnalyzer application (MassARRAY v3.4). Assays were considered optimal according to degree of clustering, absence of signal in the blanks, and reproducibility. Quality control (QC) procedures were similar to those published elsewhere [32, 33], and included 5% intraplate duplicates for each plate, 0.5% interplate duplicates, and at least two independent readers for review and interpretation of cluster plots and results. Samples with known genotypes from the Coriell Institute were also included as controls during the assay development. Samples with weak signals were repeated.

Full *CYP2D6* deletion (CYP2D6*5) analysis was conducted with a modified three-primer-based, long-range PCR using primers described by Okubo et al*.* [34] to ensure gene specificity. Genomic DNA (30 ng) was amplified in a reaction mix containing 1× Takara LA-Taq PCR buffer with 2.5 mM Mg^2+^ (Clontech, Mountain View, CA, USA), 0.5 mM dNTPs (Life Technologies, Carlsbad, CA, USA), 0.5 μM of each primer, and 0.4 units of Takara LA Taq DNA Polymerase (Clontech), in a total volume of 8 μl. Cycling included denaturation at 94 °C for 1 min, 25 cycles of 98 °C for 20 s and 70 °C for 6 min, annealing at 70 °C for 6 min, and a final hold at 4 °C. PCR products were run on 1% e-gels (Invitrogen, Carlsbad, CA, USA), and band sizes were determined against molecular weight markers to evaluate *CYP2D6* presence (4.7 kb band) or deletion (CYP2D6*5, 3.5 kb). QC procedures included use of control samples from the Coriell Institute, 5% intraplate and 0.5% interplate duplicates, as well as negative (sterile distilled water) controls.

### CYP2D6 activity score and phenotype assignment

CYP2D6 nomenclature came from the American College of Medical Genetics and Genomes (ACMG) Standards and Guidelines [35] and PharmVar (Pharmacogene Variation Database, www.Pharmvar.org/gene/CYP2D6 (Table 1). Alleles were classified into three categories based on their anticipated impact on CYP2D6 activity (normal, CYP2D6*1 and CYP2D6*2; reduced, CYP2D6*9, CYP2D6*10, and CYP2D6*41; and inactive, CYP2D6*3, CYP2D6*4, CYP2D6*5, and CYP2D6*6) [9] and assigned activity scores (AS) of 1, 0.5, and 0, respectively. The scores for the two alleles carried by each woman were summed in order to assign an overall AS which could range from 0 to 2. One exception to this is the instance of individuals carrying two IM (reduced activity) alleles. In order to distinguish between two combinations that would otherwise have had the same overall AS of 1.0, we left individuals classified as EM/PM with an AS of 1 and assigned IM/IM women an AS of 0.75 (instead of the AS of 1.0 they would otherwise have received) [26]. In this way, women were classified as: EM/EM (AS = 2), EM/IM (AS = 1.5), EM/PM (AS = 1), IM/IM (AS = 0.75), IM/PM (AS = 0.5), and PM/PM (AS = 0) [26, 36, 37]. Analyses were also conducted collapsing across some categories of the AS to generate an overall inferred phenotype. Women were classified as EM if they had at least one EM allele (i.e., AS ≥ 1), IM if they had no EM alleles and at least one IM allele (e.g., AS = 0.5–0.75), or PM if they had two PM alleles (i.e., AS = 0) [27].

### Statistical analysis

Rate ratios (RRs) and 95% confidence intervals (CIs) for the association between tamoxifen treatment and risk of CBC in our study population were estimated fitting conditional logistic regression models, stratified by CYP2D6 AS. Rate ratios can be interpreted as a relative risk (e.g., ratio of risks) of CBC in tamoxifen-treated versus untreated women in our study population. Models were adjusted for: age at first diagnosis, histology, stage, and ER status of first diagnosis, first-degree family history of breast cancer, chemotherapy for first breast cancer, radiation treatment for first breast cancer, hormonal therapy other than tamoxifen for a first breast cancer, number of full-term pregnancies before first diagnosis, and age at menarche. Because treatment for a first breast cancer could induce menopause, menopausal status and age at menopause 2 years prior to first diagnosis were used. To account for the counter-matching in WECARE I, we included a log-weight covariate offset term. For WECARE II participants who were matched in pairs (without counter-matching on radiation treatment), we assigned the value of 1 to be the offset term. We used the likelihood ratio test to test for homogeneity of treatment effect across AS/phenotypes. A sensitivity analysis was conducted restricting model fitting to Caucasian women (*N* = 1335 CBC cases and *N* = 1980 UBC controls) to address the issue of ancestral differences in genotype frequencies. All statistical analyses were conducted using SAS 9.4 (SAS Institute Inc., Cary, NC, USA).

## Results

Table 1 presents the characteristics of the women included in this analysis. The median age at diagnosis for both cases and controls was 46 years, and the majority of women were premenopausal at the time of first breast cancer diagnosis (74% of cases and 76% of controls). Just over half of all first breast cancers in cases (52%) and controls (57%) were ER-positive. Of the 793 cases with an ER-positive first primary breast cancer, 389 (49.1%) received tamoxifen. A slightly higher proportion of controls with an ER-positive breast cancer was treated with tamoxifen (53%). An additional 57 cases and 97 controls with an ER-positive first breast cancer were treated with hormonal medications other than tamoxifen (e.g., raloxifene, aromatase inhibitors). This left 839 (347 cases and 492 controls) women with an ER-positive first breast cancer who did not receive any hormonal treatment. Notably, 45 (9.6%) and 84 (15%) ER-negative cases and controls, and 31 (12.2%) and 43 (10.9%) cases and controls with unknown ER status, also received tamoxifen.

All minor allele frequencies (MAFs) were comparable to the expected frequencies in a predominantly Caucasian study population (Additional file 2). Genotyping call rates were high and ranged from 99.0 to 99.9% with intraplate and interplate concordance of 95% or greater. Although some variants were found to deviate from Hardy–Weinberg equilibrium (HWE), these results may not be meaningful given the absence of an unaffected control group.

First, we examined the effect of tamoxifen treatment for a first primary breast cancer on the risk of CBC, according to the individual CYP2D6 AS levels. Among women with AS ≥ 1, those treated with tamoxifen for a first primary breast cancer had a 20–55% lower risk of CBC (AS = 2, RR = 0.81, 95% CI 0.62–1.06; AS = 1.5, RR = 0.45, 95% CI 0.30–0.68; and AS = 1, RR = 0.55, 95% CI 0.40–0.74) relative to those not treated with tamoxifen (Table 3). Among individuals with no EM alleles and at least one PM allele, tamoxifen use was not associated with a reduction in the risk of CBC (AS = 0.5, RR = 1.08, 95% CI 0.59–1.96; and AS = 0, RR = 1.17, 95% CI 0.58–2.35) (*p* for homogeneity = 0.02). When AS categories were grouped to classify individuals based on overall phenotype (EM, AS ≥ 1; IM, AS = 0.5 or 0.75; PM, AS = 0), women with at least one fully functional (EM) allele (i.e., AS ≥ 1) treated with tamoxifen had nearly a 40% lower risk of CBC relative to those not treated with tamoxifen (RR = 0.63, 95% CI 0.51–0.78). Conversely, women classified as IM or PM (i.e., AS < 1) treated with tamoxifen were not at a lower risk of CBC (RR = 0.95, 95% CI 0.57–1.56 and RR = 1.18, 95% CI 0.59–2.37, respectively). However, these results (by AS phenotype) were not statistically significantly different (*p* for homogeneity of 0.09). Restricting the analyses to women with an ER-positive first primary breast cancer, to women who were premenopausal at first diagnosis, or to Caucasian women did not alter the results (Additional files 3, 4, and 5).

**Table 3:** Association between tamoxifen treatment and risk of CBC stratified by CYP2D6 activity score in the WECARE Study population^a^

Activity score^b^	No tamoxifen treatment			Tamoxifen treatment			
	Cases *N* (%)	Controls *N* (%)	RR (95% CI)	Cases *N* (%)	Controls *N* (%)	RR^c^ (95% CI)	*p* ^d^
2	395 (38)	549 (39)	Reference	193 (42)	300 (38)	0.81 (0.62–1.06)	0.02
1.5	192 (18)	205 (14)	Reference	65 (14)	141 (18)	0.45 (0.30–0.68)	
1	317 (30)	436 (31)	Reference	128 (28)	235 (30)	0.55 (0.40–0.74)	
0.75	30 (3)	32 (2)	Reference	20 (4)	23 (3)	0.64 (0.26–1.58)	
0.5	58 (6)	98 (7)	Reference	38 (8)	54 (7)	1.08 (0.59–1.96)	
0	57 (5)	98 (7)	Reference	21 (5)	32 (4)	1.17 (0.58–2.35)	
Phenotype^e^							
EM	904 (86)	1190 (84)	Reference	386 (83)	676 (86)	0.63 (0.51–0.78)	0.09
IM	88 (8)	130 (9)	Reference	58 (12)	77 (10)	0.95 (0.57–1.56)	
PM	57 (5)	98 (7)	Reference	21 (5)	32 (4)	1.18 (0.59–2.37)	

*CBC* contralateral breast cancer, *WECARE* Women’s Environmental Cancer and Radiation Epidemiology, *RR* rate ratio, *CI* confidence interval, *EM* extensive metabolizer, *IM* intermediate metabolizer, *PM* poor metabolizer

^a^Analysis includes 1514 CBC cases and 2203 unilateral breast cancer controls

^b^Activity score (AS) is derived from diploid phenotypes: PM/PM (AS = 0), PM/IM (AS = 0.5), IM/IM (AS = 0.75), PM/EM (AS = 1), IM/EM (AS = 1.5), and EM/EM (AS = 2)

^c^Adjusted for: age at first primary, age at menopause 2 years prior to first primary cancer, histology of first primary cancer, stage of first primary cancer, family history of breast cancer, chemotherapy at first primary cancer, radiation at first primary cancer, other hormonal therapy at first primary cancer, number of full-term pregnancies at first primary cancer, age at menarche, estrogen receptor status of first breast cancer diagnosis, and an offset term to take into account the counter-matching for radiation treatment used in WECARE I

^d^*p* value for the test that all RRs are equal across AS/phenotype categories (*p* for homogeneity)

^e^Phenotype defined as: EM, having at least one EM allele (i.e., AS ≥ 1); IM, having no EM alleles and at least one IM allele (i.e., AS = 0.5–0.75); and PM, having two PM alleles (i.e., AS = 0)

## Discussion

Prior studies have examined the association between CYP2D6 phenotype and breast cancer recurrence, recurrence-free survival, and breast cancer-specific and overall survival, with mixed results. A recent meta-analysis found a small but statistically significant increase in tumor recurrence in women who were classified as poor metabolizers (HR = 1.25, 95% CI 1.06–1.47) [38], although results were variable (and not statistically significant) when criteria for study inclusion were modified to include additional studies. The mixed findings of these prior studies [21–25] suggest that, although *CYP2D6* variation has been shown to influence tamoxifen metabolism, the impact of this variation on recurrence and survival is likely null or small [9, 27]. Indeed, current recommendations do not support routine genotyping of *CYP2D6* to guide tamoxifen treatment decisions [39]. The current study, however, is the first to address the impact of this genetic variation on risk of CBC following tamoxifen treatment for the first primary breast cancer.

The results of this study suggest that the CYP2D6 phenotype could modify the association between tamoxifen treatment for a first primary breast cancer and risk of CBC. While women with CYP2D6 AS ≥ 1 (i.e., EM) who were treated with tamoxifen had a lower risk of CBC relative to those not treated with tamoxifen (RR = 0.63, 95% CI 0.51–0.78), women classified as having AS < 1 (i.e., IM or PM) who were treated with tamoxifen were not at lower risk of CBC (RR = 0.95, 95% CI 0.57–1.56 and RR = 1.18, 95% CI 0.59–2.37, respectively). Although these results are not statistically significantly different (*p* for homogeneity = 0.09), the overall trend appeared to be consistent. The exception is the finding for EM/EM (AS = 2), where RR = 0.81 (Table 3); this appears to deviate from the otherwise monotonic trend of decreasing RR associated with tamoxifen treatment, with increasing AS.

Like others [26], we made a slight modification to our calculation of the AS for individuals carrying two IM (i.e., reduced activity) alleles, where we provided a distinction between individuals classified as EM/PM (AS = 1), IM/IM (AS = 0.75), and IM/PM (AS = 0.5). Coding the AS this way allowed us to distinguish between different IM diplotypes, which have been shown to have variable levels of enzyme activity [36]. In particular, there is some evidence that CYP2D6*10, a reduced activity allele, may have activity that is closer to 0 than 1 [9]. These categories were then combined in the overall phenotype analysis.

Both randomized controlled clinical trials [3, 40] and observational studies [41–43], including those from our group [5, 6, 44], have found a protective effect of tamoxifen with respect to risk of CBC. Some studies have found that this effect is limited to women diagnosed with ER-positive breast cancer [40], while others have not [5, 6, 41]. We have previously reported that tamoxifen treatment was associated with approximately a 27% lower risk of CBC, with no evidence that the association differed by ER status of the first primary breast cancer [6].

In our study, a relatively large proportion (41%) of women with ER-positive first breast cancers did not receive hormonal therapy of any kind, while some women with ER-negative breast cancer did (18%). This may have happened because approximately 20% of women included in this study were diagnosed with a first primary breast cancer prior to 1989 (Table 1), a time when tamoxifen was given as a treatment regardless of ER status. Notably, just as tamoxifen is used as a chemopreventive agent in women at high risk of developing a first breast cancer (e.g., *BRCA* mutation carriers) [45], tamoxifen treatment for a first breast cancer can be seen to serve two functions; first, to treat the diagnosed cancer; and second, to prevent a second, independent breast cancer (i.e., CBC).

A major and unique strength of this study is our ability to investigate the impact of the CYP2D6 phenotype specifically on the risk of CBC. This was made possible given our use of a retrospective, multicenter, population-based design, which allowed for the inclusion of a large number of women diagnosed with CBC. Another key strength was the availability of detailed treatment histories from medical records, including both tamoxifen treatment as well as other treatments that were included as covariates in the analysis. This study also has some limitations. Information regarding use of other medications (beyond those used for treatment of a first breast cancer) was not collected. Consequently, we were not able to account for drugs that have been observed to inhibit CYP2D6 (e.g., SSRIs), although evidence to date suggests that any possible effects are likely quite modest [46]. We also had no information on *CYP2D6* copy number variation and therefore were not able to identify ultra-rapid metabolizers within our study population. In addition, the WECARE Study predominantly consists of Caucasian women, limiting the generalizability of our results somewhat. However, although the *CYP2D6* variant frequency and corresponding inferred phenotypes have been shown to vary by race/ethnicity [47], their impact on metabolite concentration does not [26].

Aromatase inhibitors are now the first-line hormonal treatment for postmenopausal women with ER-positive breast cancer [48], while tamoxifen treatment remains the first-choice treatment for premenopausal women. Our study includes women who were younger than 55 years of age at the time of first diagnosis, so that the majority (~ 75%) of study participants were premenopausal at the time of first breast cancer diagnosis and treatment. Thus, these results are generalizable to women who are most likely to be prescribed tamoxifen under current standards of care. With a more precise identification of women in whom tamoxifen treatment is unlikely to effectively reduce their risk of CBC, alternative therapies can then be offered (e.g., ovarian oblation plus aromatase inhibitors in premenopausal women) [49]. This would be particularly important for women known to be at an increased risk of CBC (e.g., women with a family history of CBC) [50]. Since our findings suggest that CYP2D6 phenotype may impact the effectiveness of tamoxifen for prevention of second primary breast cancers, it may also be relevant with regards to the use of tamoxifen as a chemopreventive agent among high-risk women. To our knowledge only a single study has examined the impact of the CYP2D6 phenotype on breast cancer prevention with tamoxifen, and no association between the CYP2D6 phenotype and ER-positive breast cancer occurrence was observed [51].

## Conclusions

Women frequently overestimate their risk of CBC, leading to increasing rates of contralateral prophylactic mastectomy. Overall, this study provides early evidence that the CYP2D6 phenotype may contribute to some of the observed variability in the impact of tamoxifen treatment for a first breast cancer on the risk of developing CBC. Identifying factors that can influence a woman’s risk of developing CBC will inform discussions between patient and physician, help guide treatment decisions, and strengthen evidence-based risk communication.

## Additional files

Additional file 1: CYP2D6 polymorphisms tested, primer sequences, and PCR conditions for genotyping. (XLSX 10 kb)

Additional file 2: *CYP2D6* variant details by case–control status. (DOCX 27 kb)

Additional file 3: Association between tamoxifen treatment and risk of CBC stratified by CYP2D6 activity score in women with ER-positive first breast cancer in the WECARE Study population. (DOCX 16 kb)

Additional file 4: Association between tamoxifen treatment and risk of CBC stratified by CYP2D6 activity score in women who are premenopausal at first diagnosis in the WECARE Study population. (DOCX 17 kb)

Additional file 5: Association between tamoxifen treatment and risk of CBC stratified by CYP2D6 activity score in Caucasian women in the WECARE Study population (DOCX 16 kb)

## Abbreviations

AS: Activity score
CBC: Contralateral breast cancer
CI: Confidence interval
EM: Extensive metabolizer
ER: Estrogen receptor
HWE: Hardy–Weinberg equilibrium
IM: Intermediate metabolizer
MAF: Minor allele frequency
PM: Poor metabolizer
QC: Quality control
RR: Rate ratio
SEER: Surveillance Epidemiology and End Results
SNP: Single nucleotide polymorphism
UBC: Unilateral breast cancer
WECARE: Women’s Environmental Cancer and Radiation Epidemiology
