# Supplementary material for: CYP2D6 phenotype, tamoxifen, and risk of contralateral breast cancer in the WECARE Study
Source: Breast Cancer Res. 2018 Dec 10;20:149. doi: 10.1186/s13058-018-1083-y (PMC6288916; doi:10.1186/s13058-018-1083-y)
Supplement: Supplementary file 2 — CYP2D6 variant details by case–control status. (DOCX 27 kb) [file 13058_2018_1083_MOESM2_ESM.docx]

**Additional file 2: *CYP2D6* variant details by case-control status.**

| **Variant** | **CBC Cases**  **N=1,514**  **N(%)** | **UBC Controls**  **N=2,203**  **N(%)** | **MAF^1^** | **MAF in EUR^2^** | **HWE^3^** |
| --- | --- | --- | --- | --- | --- |
| **rs16947** |  |  |  |  |  |
| CC | 689 (46) | 1,008 (46) | 0.34 | 0.34 | <0.0001 |
| CT | 604 (40) | 884 (40) |  |  |  |
| TT | 207 (14) | 286 (13) |  |  |  |
| Missing | 14 (1) | 25 (1) |  |  |  |
| **rs1135840** |  |  |  |  |  |
| GG | 529 (35) | 742 (34) | 0.43 | 0.45 | 0.01 |
| GC | 681 (45) | 1015 (46) |  |  |  |
| CC | 300 (20) | 431 (20) |  |  |  |
| Missing | 4 (0) | 15 (1) |  |  |  |
| **rs35742686** |  |  |  |  |  |
| AA | 1,474 (97) | 2,128 (97) | 0.01 | 0.02 | 0.06 |
| A.DEL | 36 (2) | 68 (3) |  |  |  |
| DEL | 1 (0) | 2 (0) |  |  |  |
| Missing | 3 (0) | 5 (0) |  |  |  |
| **rs3892097** |  |  |  |  |  |
| GG | 993 (66) | 1,418 (64) | 0.19 | 0.19 | 0.64 |
| GA | 451 (30) | 685 (31) |  |  |  |
| AA | 59 (4) | 88 (4) |  |  |  |
| Missing | 11 (1) | 12 (1) |  |  |  |
| **rs5030655** |  |  |  |  |  |
| TT | 1,493 (99) | 2,163 (98) | 0.01 | 0.02 | <0.0001 |
| T_ | 16 (1) | 33 (2) |  |  |  |
| DEL | 2 (0) | 2 (0) |  |  |  |
| Missing | 3 (0) | 5 (0) |  |  |  |
| **rs5030656** |  |  |  |  |  |
| AAG | 1,432 (95) | 2,094 (95) | 0.03 | 0.03 | 0.16 |
| AAG.DEL | 80 (5) | 105 (5) |  |  |  |
| DEL | 2 (0) | 3 (0) |  |  |  |
| Missing | 0 (0) | 1 (0) |  |  |  |
| **rs1065852** |  |  |  |  |  |
| CC | 917 (61) | 1,324 (60) | 0.23 | 0.20 | 0.001 |
| CT | 482 (32) | 720 (33) |  |  |  |
| TT | 100 (7) | 143 (6) |  |  |  |
| Missing | 15 (1) | 16 (1) |  |  |  |
| **rs28371725** |  |  |  |  |  |
| GG | 1,261 (83) | 1,862 (85) | 0.09 | 0.09 | <0.0001 |
| GA | 222 (15) | 303 (14) |  |  |  |
| AA | 30 (2) | 37 (2) |  |  |  |
| Missing | 1 (0) | 1 (0) |  |  |  |

Abbreviations: CBC: contralateral breast cancer, UBC: unilateral breast cancer, MAF: minor allele frequency, EUR: European ancestry, HWE: Hardy-Weinberg Equilibrium.

^1^MAF from the WECARE Study population (cases and controls combined), which is predominantly Caucasian (~90%)

^2^MAF for populations with European ancestry (EUR) from <https://www.ensemble.org>, accessed October 2017.

^3^HWE in UBC controls
