# Supplementary material for: CYP2D6 phenotype, tamoxifen, and risk of contralateral breast cancer in the WECARE Study
Source: Breast Cancer Res. 2018 Dec 10;20:149. doi: 10.1186/s13058-018-1083-y (PMC6288916; doi:10.1186/s13058-018-1083-y)
Supplement: Supplementary file 5 — Association between tamoxifen treatment and risk of CBC stratified by CYP2D6 activity score in Caucasian women in the WECARE Study population (DOCX 16 kb) [file 13058_2018_1083_MOESM5_ESM.docx]

**Additional file 5: Association between tamoxifen treatment and risk of CBC stratified by CYP2D6 Activity Score in the WECARE Study Population—Non-Hispanic Whites.**

| **Activity Score^a^** | **No Tamoxifen Treatment** | | | **Tamoxifen Treatment** | | | |
| --- | --- | --- | --- | --- | --- | --- | --- |
|  | **Cases**  **N (%)** | **Controls**  **N (%)** | **RR**  **(95% CI)** | **Cases**  **N (%)** | **Controls**  **N (%)** | **RR^c^**  **(95% CI)^c^** | **p-het** |
| 2 | 330 (35) | 487 (37) | Ref | 162 (41) | 242 (36) | 0.88 (0.66, 1.19) | 0.02 |
| 1.5 | 169 (18) | 185 (14) | Ref | 53 (13) | 117 (17) | 0.46 (0.29, 0.71) |  |
| 1 | 295 (32) | 411 (32) | Ref | 118 (30) | 215 (32) | 0.56 (0.41, 0.78) |  |
| 0.75 | 24 (3) | 26 (2) | Ref | 12 (3) | 16 (2) | 0.56 (0.19, 1.59) |  |
| 0.5 | 57 (6) | 97 (7) | Ref | 35 (9) | 50 (7) | 1.10 (0.60, 2.04) |  |
| 0 | 56 (6) | 94 (7) | Ref | 19 (5) | 31 (5) | 1.10 (0.54, 2.24) |  |
| **Activity Score Phenotype^b^** |  |  |  |  |  |  |  |
| EM | 794 (85) | 1083 (83) | Ref | 333 (83) | 574 (86) | 0.66 (0.53, 0.83) | 0.19 |
| IM | 81 (9) | 123 (9) | Ref | 47 (12) | 66 (10) | 0.96 (0.56, 1.63) |  |
| PM | 56 (6) | 94 (7) | Ref | 19 (5) | 31 (5) | 1.11 (0.54, 2.26) |  |

Abbreviations: RR: Rate Ratio, CI: confidence interval, EM: extensive metabolizer, IM: intermediate metabolizer, PM: poor metabolizer, AS: Activity Score

^a^Activity Score is derived from diploid phenotypes: PM/PM (0), PM/IM (0.5), IM/IM (0.75), PM/EM (1), IM/EM (1.5), EM/EM (2)

^b^Activity Score Phenotype defined as: EM: having at least one EM allele (ie., AS≥1), IM: having no EM alleles and at least one IM allele (i.e., AS = 0.5-0.75), PM: having two PM alleles (i.e., AS = 0)

^c^Adjusted for age at first primary, age at menopause two years prior to first primary, histology of first primary, stage of first primary, family history of breast cancer, chemotherapy at first primary, radiation at first primary, other hormonal therapy for a first primary, number of full term pregnancies at first primary, age at menarche, ER status of first breast cancer diagnosis.
